# Pareto Design of State Feedback Tracking Control of a Biped Robot via Multiobjective PSO in Comparison with Sigma Method and Genetic Algorithms: Modified NSGAII and MATLAB's Toolbox

**DOI:** 10.1155/2014/303101

**Published:** 2014-01-27

**Authors:** M. J. Mahmoodabadi, M. Taherkhorsandi, A. Bagheri

**Affiliations:** ^1^Department of Mechanical Engineering, Sirjan University of Technology, Sirjan, Iran; ^2^Department of Mechanical Engineering, University of Texas at San Antonio, San Antonio, TX 78249, USA; ^3^Department of Mechanical Engineering, Faculty of Engineering, The University of Guilan, Rasht, Iran

## Abstract

An optimal robust state feedback tracking controller is introduced to control a biped robot. In the literature, the parameters of the controller are usually determined by a tedious trial and error process. To eliminate this process and design the parameters of the proposed controller, the multiobjective evolutionary algorithms, that is, the proposed method, modified NSGAII, Sigma method, and MATLAB's Toolbox MOGA, are employed in this study. Among the used evolutionary optimization algorithms to design the controller for biped robots, the proposed method operates better in the aspect of designing the controller since it provides ample opportunities for designers to choose the most appropriate point based upon the design criteria. Three points are chosen from the nondominated solutions of the obtained Pareto front based on two conflicting objective functions, that is, the normalized summation of angle errors and normalized summation of control effort. Obtained results elucidate the efficiency of the proposed controller in order to control a biped robot.

## 1. Introduction

Biped robots are one of crucial kinds of mobile robots since they are the most similar robots to humans and have the capabilities the same as humans, such as walking, speaking, and communicating [1–3]. Undeniably, they will be utilized in industry as an alternative to the skilled workforce doing high-risk activities in the near future. To control robots for a variety of challenging tasks, researchers have used efficient robust controllers, such as practical velocity tracking control [4], dynamic state feedback control [5], fuzzy PD control [6], and neural network control [7]. Specifically, Solís-Perales and Peón-Escalante [8] used robust adaptive tracking control for a class of robot manipulators having model uncertainties. Indeed, they utilized a linearizing-like control feedback and a high-gain estimator for a model with four unknown parameters, that is, system parameters, nonlinear terms, external perturbations, and the friction effects in each robot joint. Akbari et al. [9] employed a fuzzy TSK controller to control a rotary flexible joint manipulator modeled by the use of a solenoid nonlinear spring. It has been illustrated that state feedback controllers are effectual controllers in the aspect of having acceptable tracking error and control effort. In particular, Montagner and Ribas [10] used state feedback control for tracking sinusoidal references to reject disturbances affecting the plant. They utilized three techniques, linear quadratic regulator, the pole placement, and the *H*_*∞*_ control to control uninterruptible power supply systems. Chang and Fu [11] proposed dynamic state feedback formation control for achieving the realization of the multirobot formation system with respect to the problem of dilation of a formation shape and stabilization issue in a nonholonomic system simultaneously. Oliveira et al. [12] proposed a state feedback technique based on the modification of states transition matrix and used Genetic Algorithm (GA) to eliminate the trial and error process. Solihin et al. [13] utilized state feedback control for tracking control of a flexible link manipulator. They also employed the adopting particle swarm optimization algorithm to omit the tedious trial and error process of determining heuristic parameters in state feedback control.

Particle swarm optimization (PSO) is regarded as a smart optimization evolutionary and simulating algorithm introduced by Kennedy and Eberhart [14] having outstanding qualities, such as a high convergence rate and the capability of solving complicated optimization problems. PSO was derived from human behavior and animal behavior and it is easy to implement owing to having few parameters to adjust and special characteristic of memory [15]. In that respect, it has been successfully promoted by a number of researchers and applied in a wide range of scientific fields, to name it a few, control, electronics, robotics, and economics [16–35]. Although having many excellent qualities, PSO suffers premature convergence due to losing of diversity. To solve the premature convergence, Wang et al. [36] proposed a hybrid PSO algorithm utilizing a diversity improving mechanism and neighborhood search strategies to reach a trade-off between exploration and exploitation abilities. Zhou et al. [37] employed the concept of the mutation by using random factors to augment the global search ability of particle swarm optimization. In order to enhance the search capabilities of PSO, it has been combined with other optimization algorithms. Idoumghar et al. [38] utilized particle swarm optimization combined with simulated annealing algorithms to avoid local optimal solutions and premature convergence of PSO. Qian et al. [39] combined particle swarm optimization with the simplex method to adopt a hierarchical and cooperative regime of global search and local search to optimize the objective function. Liu and Yang [40] utilized particle swarm optimization combined with the Nelder-Mead simplex method to enhance its potential for rapid convergence.

Undeniably, the objective functions of practical engineering problems conflict with each other. Hence, designers prefer to use multiobjective optimization algorithms to regard all objective functions based on the design criteria. To this end, several approaches, such as dynamic neighborhood PSO [41], dominated tree [42], sigma method [43], vector evaluated PSO [44], dynamic multiple swarms [45], dynamic population size, and adaptive local archives [46], have been proposed to develop the PSO algorithm to deal with multi-objective optimization problems. By comparing the above-mentioned techniques, it can be concluded that the main difference among those approaches is the leader selection techniques. When all particles are updated in the current iteration, some of nondominated solutions are similar in the objective function space. Keeping all of them in the archive needs a great deal of space. Moreover, it will also preclude uniform diversity of nondominated solutions. To overcome these drawbacks, a fuzzy approach is utilized in this study.

Recently, Hassanzadeh and Mobayen [47] utilized the genetic algorithm, particle swarm optimization, and ant colony to balance the pendulum in the rotational inverted position. They demonstrated the efficiency of the proposed controller with respect to parameter variations, noise effects, and load disturbances. Wang and Guan [48] successfully utilized optimal control based on particle swarm optimization for a parallel hybrid hydraulic excavator. Gao et al. [49] used a typical fractional order PID control strategy based upon the improved multi-objective differential evolutionary algorithm for the gun control system.

This investigation develops significantly authors' previous work [50, 51] as follows: Taherkhorsandi et al. [50] used a linear quadratic tracking controller to control a biped robot stepping on a flat surface; however, a nonlinear state feedback tracking controller is used here to control the biped robot walking on slope. Optimal state feedback tracking control using a multi-objective particle swarm optimization algorithm in comparison to three prominent optimization algorithms, modified NSGAII, Sigma method, and MATLAB's Toolbox MOGA is used here to design the parameters of the proposed controller, while, sliding mode control based upon a particle swarm optimization algorithm is utilized in [51] to control the biped robot.

## 2. The Dynamics of the Biped Robot

**Figure 1:**
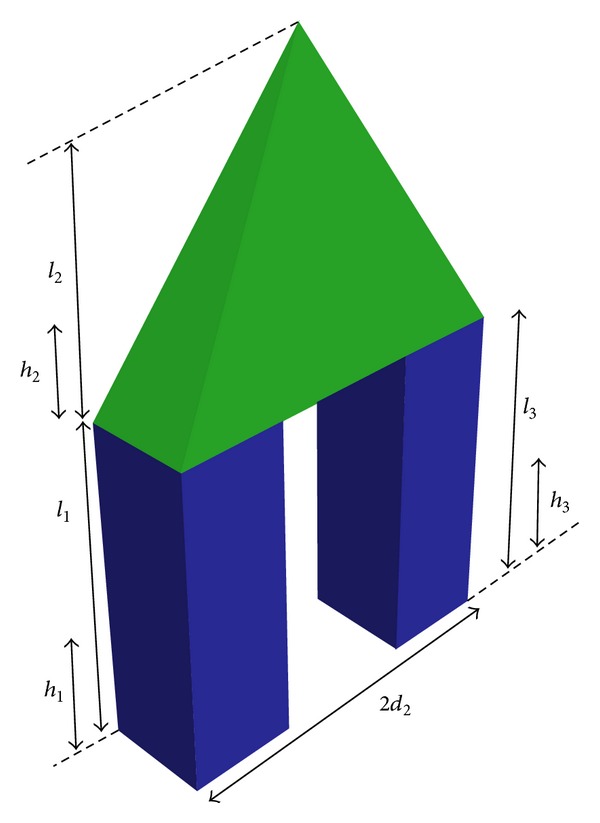
The parameters of the robot based on the anthropometric table.

In the present study, a biped robot is walking in the lateral plane on slope [50]. To model this robot, a three-link planar is used according to Figure 1. The first link represents the stance leg on the ground, the second link signifies the head, arms, and trunk, and the third link is the swing leg. In fact, those links move freely in the lateral plane. The parameters of the biped robot are obtained from Table 1 for a humanoid robot having 171 (cm) height and 74 (kg) weight [53]. The distance between two legs of the model (2*d*_2_) equals 32.7 (cm).

To obtain the dynamic equations of the biped robot, Newton-Euler method is employed to derive the dynamic equations of the model. *θ*_1_, *θ*_2_, and *θ*_3_ are the angles between the first, second, and third links and assumed vertical line of these links, correspondingly. Hence, the equations of the model for *θ*_1_, *θ*_2_, and *θ*_3_ are

(1) I1θ¨1=u1−u2+h1m1gsin⁡θ1+l1sinθ1g(m2+m3)+h1m1(−h1θ¨1)+l1m2[−θ¨1l1+θ¨2{d2sin⁡(θ2−θ1)−h2cos⁡⁡(θ2−θ1)}+θ˙22{d2cos⁡⁡(θ2−θ1)+h2sin⁡(θ2−θ1)}]+l1m3[−θ¨1l1+θ2¨{2d2sin⁡(θ2−θ1)−θ3(l2−h3)cos⁡⁡(θ3−θ1)}+θ˙22  2d2cos⁡⁡(θ2−θ1)+θ˙32(l3−h3)sin⁡(θ3−θ1)],I2θ¨2=u2−u3+m2d2gcos⁡θ2+m2h2gsinθ2+2m3d2gcos⁡θ2−θ¨1[cos⁡⁡(θ2−θ1)m2h2l1−sin⁡(θ2−θ1)m32d2l1−sin⁡(θ2−θ1)m2d2l1]+θ˙12[sin⁡(θ1−θ2)m2l1h2−cos⁡⁡(θ2−θ1)2m3d2l1−cos⁡⁡(θ2−θ1)m2d2l1]+θ¨2[−m2d22−4m3d22−m2h22]+θ¨3[2m3d2(l3−h3)sin⁡(θ2−θ3)]−θ˙32[2m3d2(l3−h3)cos⁡⁡(θ2−θ3)],I3θ¨3=(l3−h3)m3gsin⁡θ3+u3−m3(l3−h3)l1cos⁡⁡(θ3−θ1)θ¨1+m3(l3−h3)l1sin⁡(θ1−θ3)θ˙12+2d2m3(l3−h3)sin⁡(θ2−θ3)θ¨2+2d2m3(l3−h3)cos⁡⁡(θ3−θ2)θ¨2−m3(l3−h3)2θ¨3.

In this study, the biped robot passes two phases, double support phase (DSP) and single support phase (SSP). In DSP, both feet are on the ground; however, in SSP, the biped robot has one contact surface with the floor. The time of DSP is regarded as 20 percent of the whole time [54]. Moreover, the swing foot trajectory having the first-order continuity is generated and it would maintain the zero moment point on the inside of the support polygon. Then, the inverse kinematics is employed to acquire the desired trajectories of the joints. The desired trajectories should have first-order and second-order continuity. The first-order derivative continuity guarantees the smoothness of the joint velocity, while the second-order continuity guarantees the smoothness of the acceleration or torque on the joints.

## 3. Multiobjective Particle Swarm Optimization

### 3.1. Particle Swarm Optimization

Particle swam optimization is a smart evolutionary and simulating algorithm motivated by the simulation of social behavior instead of survival of the fittest [14]. At first, PSO was proposed to tune weight functions in neural networks [55]; however, it is now utilized as an effectual optimization algorithm where the decision variables are real numbers [56, 57]. The candidates for solutions are named particles and the position of them changes based on every particle's experience and neighbors (velocity). Indeed, each candidate solution is associated with a velocity [58]. The governing equations for particles are as follows:

(2) xi→(t+1)=xi→(t)+vi→(t+1),vi→(t+1)=Wvi→(t)+C1r1(x→pbesti−xi→(t))+C2r2(x→gbest−xi→(t)),

where $\overset{\to }{x_{i}}(t)$ is the position of particle *i* and $\overset{\to }{v_{i}}(t)$ presents the velocity of particle *i*, at time step *t*. *W* is the inertia weight utilized to control the impact of the previous history of velocities on the current velocity of a given particle. *C*_1_ is the cognitive learning factor illustrating the attraction which a particle has toward its own success. *C*_2_ is the social learning factor representing the attraction which a particle has toward the success of the entire swarm. *r*_1_, *r*_2_ ∈ [0,1] are random values. $\overset{\to }{x}{}_{g\text{best}}$ is the position of the best particle of the entire swarm and $\overset{\to }{x}{}_{p{\text{best}}_{i}}$ is the best personal position of the particle *i*. Inertia weight is used to tune the global and local search ability and it has qualities which are reminiscent of the temperature parameter in the simulated annealing [58]. It is crucial to note that the large inertia weight makes a global search straightforward; however, the small inertia weight facilitates a local search. In that respect, by changing the inertia weight dynamically, the search ability is dynamically adjusted. Eberhart and Kennedy [59] illustrated that decreasing inertia weight linearly over the iterations enhances the performance of PSO. Particles are permitted to move around their best personal position ($\overset{\to }{x}{}_{p{\text{best}}_{i}}$) by using a large value of *C*_1_ and a small value of *C*_2_. Moreover, particles converge to the best particle of the whole swarm ($\overset{\to }{x}{}_{g\text{best}}$) by utilizing a small value of *C*_1_ and a large value of *C*_2_. By regarding the above-mentioned results, it was observed that best solutions were acquired when *C*_1_ is decreased linearly and *C*_2_ is increased linearly over the iterations [57]. In this study, the linear formulation for inertia weight and learning factors are employed as follows:

(3) $$\begin{array}{c} W=W_{1}-\left(W_{1}-W_{2}\right)\times \left(\frac{t}{\text{maximum}\;\text{iteration}}\right),\\ C_{1}=C_{1i}-\left(C_{1i}-C_{1f}\right)\times \left(\frac{t}{\text{maximum}\;\text{iteration}}\right),\\ C_{2}=C_{2i}-\left(C_{2i}-C_{2f}\right)\times \left(\frac{t}{\text{maximum}\;\text{iteration}}\right), \end{array}$$

where *W*_1_ and *W*_2_ are the initial and final values of the inertia weight, respectively. *C*_1*i*_ and *C*_2*i*_ are the initial values of the learning factors *C*_1_ and *C*_2_, respectively. *C*_1*f*_ and *C*_2*f*_ are the final values of the learning factors *C*_1_ and *C*_2_, respectively. *t* is the current iteration number and maximum iteration is the maximum number of allowable iterations.

### 3.2. Multiobjective Particle Swarm Optimization

Multi-objective optimization is gaining a vector of decision variables satisfying constraints to give acceptable values to all objective functions [60]. It involves the vector of design variables and the vector of objective functions. Multi-objective minimization based on the Pareto technique can be conducted using some definitions [61].

The definition of Pareto optimality: a point X* ∈ *Ω* (Ω is a feasible region in *R* ^*n*^) is Pareto optimal (minimal) if and only if there is not *X* ∈ *Ω* which is dominant over X*. Alternatively, it can be readily restated as ∀*X* ∈ *Ω*, *X* ≠ X*, ∃*i* ∈ {1,2,…, *k*} : *f*_*i*_(X*) < *f*_*i*_(*X*). 

The definition of Pareto dominance: a vector $\vec{U}=[u_{1},u_{2},\ldots ,u_{n}]$, is dominant over the vector $\vec{V}=[v_{1},v_{2},\ldots ,v_{n}]$ (denoted by $\vec{U}≺\vec{V}$) if and only if ∀*i* ∈ {1,2,…, *n*}, *u*_*i*_ ≤ *v*_*i*_∧∃*j* ∈ {1,2,…, *n*} : *u*_*j*_ < *v*_*j*_.

The definition of Pareto front: for a given multi-objective optimization problem, the Pareto front PT* is a set of vectors of objective functions gained by utilizing the vectors of decision variables in the Pareto set P*; that is, PT* = {*F*(*X*) = (*f*_1_(*X*), *f*_2_(*X*),…, *f*_*k*_(*X*)) : *X* ∈ P*}.

The definition of Pareto set: for a given multi-objective optimization problem, a Pareto set P* is a set in the decision variable space consisting of all the Pareto optimal vectors P* = {*X* ∈ *Ω* | ∄*X*′ ∈ *Ω* : *F*(*X*′)≺*F*(*X*)}. Indeed, the Pareto front PT* is a set of the vectors of objective functions mapped from P*.

In multi-objective particle swarm optimization, a set of different leaders is devoted to each particle and one of the leaders could be chosen to update the position of a particle; however, one leader is utilized to update the positions of particles in single-objective optimization problems. In elaboration, one leader should be selected as *g*best in order to update the position of each particle and enhance the convergence and diversity of solutions. To this end, a leader selection approach based on density measures is utilized. A neighborhood radius *R*_neighborhood_ is defined for all nondominated solutions. Two nondominated solutions are neighbors if the Euclidean distance measured in the objective domain between them is fewer than *R*_neighborhood_. Hence the particle having fewer neighbors is preferred as a leader. On the other hand, by choosing an appropriate approach to find $\overset{\to }{x}{}_{p{\text{best}}_{i}}$ for particle *i*th, the diversity within the swarm is maintained. Here, Sigma method is utilized to find the best personal positions of particles; however, this method was proposed to find the best local guides [43]. When *σ*_*i*_ and *σ*_*j*_ are devoted to each particle in the population and archive, respectively, the particle *k* in the archive is chosen as the best personal position of the particle *i* where the distance between *σ*_*i*_ and *σ*_*k*_ is minimized. By regarding a two-objective space, the parameter *σ* is defined as follows:

(4) $$\begin{array}{c} \sigma =\frac{f_{1}^{2}-f_{2}^{2}}{f_{1}^{2}+f_{2}^{2}}. \end{array}$$

In the present study, a turbulence operator is employed to find more appropriate positions and avoid being trapped in a local minimum. *N* particles in the population are randomly chosen to add the turbulence factor to their position vectors:

(5) $$\begin{array}{c} {\vec{x}}_{i}\left(t\right)=\overset{\to }{x}{}_{\min}\left(t\right)+\text{rand}\times \left(\overset{\to }{x}{}_{\max}-{\overset{\to }{x}}_{\min}\right), \end{array}$$

where rand is a random number generated uniformly in the interval [0,1]. $\vec{x}{}_{\max}$ and $\vec{x}{}_{\min}$ are upper bound and lower bound of the search space. In this paper, *N* = *P*_*m*_ × number  of  particles where *P*_*m*_ is the probability of the turbulence operator and set at 5/*t*.

## 4. The Pareto Design of State Feedback Control

The stages of state feedback control are designed and constructed step by step as follows. To control the system, the state variable vector is chosen as $\left(\begin{array}{c} x_{1}\\ x_{2}\\ x_{3}\\ x_{4}\\ x_{5}\\ x_{6} \end{array}\right)=\left(\begin{array}{c} \theta_{1}\\ {\dot{\theta }}_{1}\\ \theta_{2}\\ {\dot{\theta }}_{2}\\ \theta_{3}\\ {\dot{\theta }}_{3} \end{array}\right)$. And the errors could be defined as

(6) $$\begin{array}{c} E_{p}=\theta_{dp}-\theta_{p}\;\left(p=1,2,3\right),\\ {\dot{E}}_{q}={\dot{\theta }}_{dq}-{\dot{\theta }}_{q}\;\left(q=1,2,3\right). \end{array}$$

Finally, the control effort is obtained by

(7) $$\begin{array}{c} u=\sum_{p=1}^{3}K_{p}E_{p}+\sum_{q=1}^{3}K_{q}^{\prime }{\dot{E}}_{q}, \end{array}$$

where *K*_*p*_ and *K*_*q*_′  (*p*  and  *q* = 1,2, 3) are the state feedback parameters.

The proposed method is used to find the proper state feedback parameters and remove the tedious and repetitive trial and error process. Furthermore, the results are compared with three prominent algorithms. The performance of a controlled closed loop system is usually assessed by a variety of goals [62]. In this study, the normalized summation of angles errors and normalized summation of control effort are regarded as the objective functions. These objective functions are minimized at the same time. The vector [*K*_1_, *K*_2_, *K*_3_, *K*_1_′, *K*_2_′, *K*_3_′] is the vector of selective parameters of state feedback control. These are positive constants. The normalized summation of angles errors and normalized summation of control effort are functions of this vector's components. In this regard, by choosing various amounts of the selective parameters, changes occur in the normalized summation of angles errors and normalized summation of control effort. This is an optimization problem with two objective functions (the normalized summation of angles errors and normalized summation of control effort) and six decision variables (*K*_1_, *K*_2_, *K*_3_, *K*_1_′, *K*_2_′, *K*_3_′). The regions of the selective parameters are

(8) $$\begin{array}{c} -200<K_{1},\;\;K_{2}^{\prime }<0,\;\;-10000<K_{2}<-5000,\\ -200<K_{3}<-100,\;\;-10000<K_{1}^{\prime }<-4000,\\ -1000<K_{3}^{\prime }<-10. \end{array}$$

**Figure 2:**
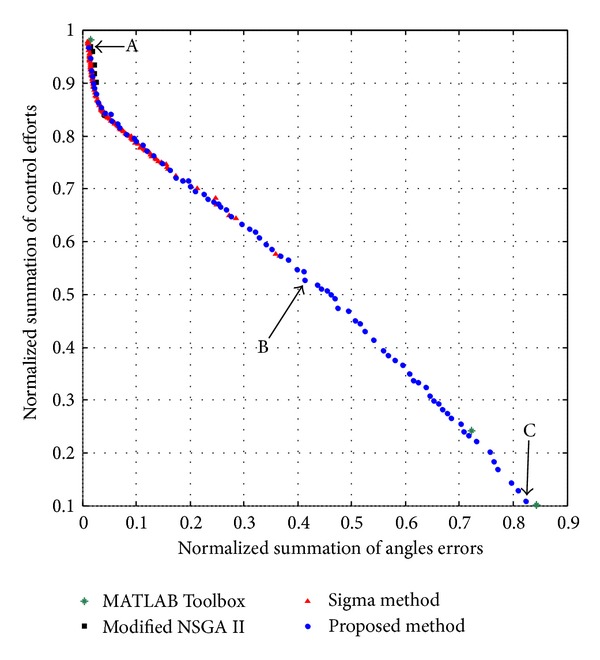
The obtained Pareto fronts by using Sigma method [43], modified NSGAII [52], MATLAB's Toolbox MOGA, and the proposed algorithm regarding the optimal control design of the biped robot.

The feasibility and efficiency of the proposed multi-objective algorithm are assessed in comparison with Sigma method [23], modified NSGAII [41], and MATLAB Toolbox MOGA. The Pareto front of this multi-objective problem is shown in Figure 2. The swarm size is 10 and the maximum iteration equals 500. The term ${\vec{v}}_{i}(t)$ is limited to the range [−*v*_ave_, +*v*_ave_] in which *v*_ave_ = (*x*_max⁡_ − *x*_min⁡_)/2. While the velocity violates this range, it will be multiplied by a random number between [0,1]. *E*_constant_^  ^ and *R*_neighborhood_^  ^ are set at 25 and 0.02, respectively. Over iteration, the inertia weight *W* is linearly decreased from *W*_1_ = 0.9 to *W*_2_ = 0.4, *C*_1_ is linearly decreased from *C*_1*i*_ = 2.5 to *C*_1*f*_ = 0.5, and *C*_2_ is linearly increased from *C*_2*i*_ = 0.5 to *C*_2*f*_ = 2.5. 

**Figure 3:**
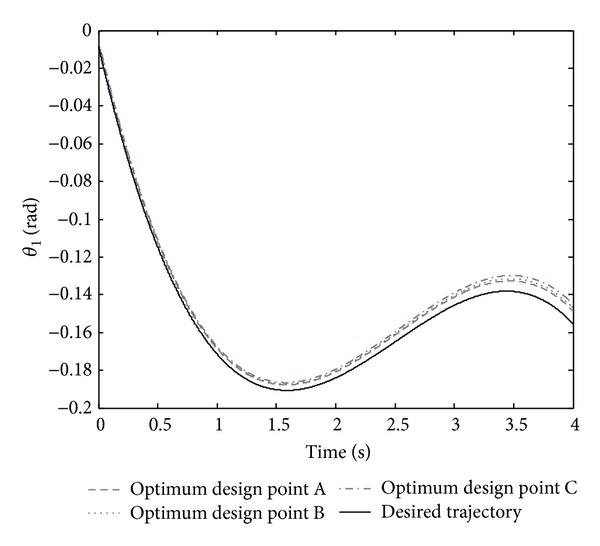
The desired trajectory of *θ*_1_ and its tracking trajectory for the optimum design points A, B, and C shown in the Pareto front.

**Figure 4:**
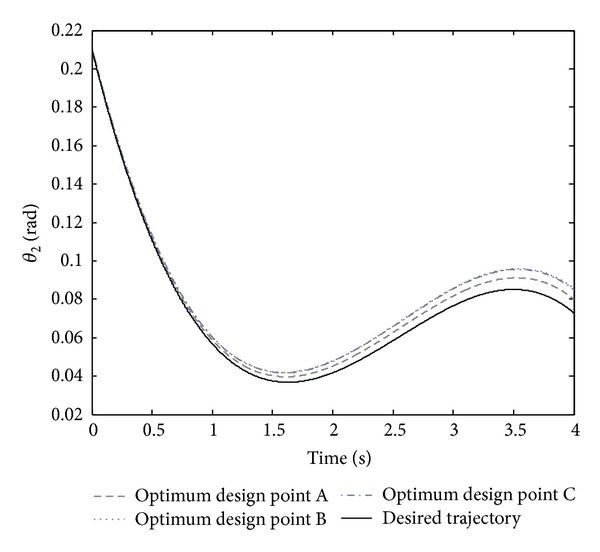
The desired trajectory of *θ*_2_ and its tracking trajectory for the optimum design points A, B, and C shown in the Pareto front.

**Figure 5:**
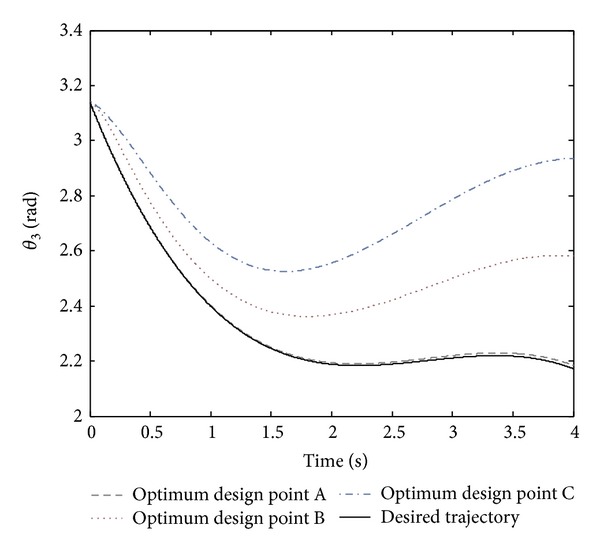
The desired trajectory of *θ*_3_ and its tracking trajectory for the optimum design points A, B, and C shown in the Pareto front.

By regarding Figure 2, all the optimal points in the Pareto front are nondominated and could be selected to design the controller. However, it is crucial to note that selecting a better amount of any objective function causes a worse amount of another objective. In the Pareto front, there are three crucial points, A, B, and C. By regarding both objective functions of the normalized summation of angles errors and normalized summation of control effort, point B could be the trade-off optimum choice. Moreover, design variables and objective functions corresponding to the optimum design points A, B, and C are illustrated in Table 2. The real tracking trajectories and phase planes of the optimum design points A, B, and C are shown in Figures 3, 4, and 5.

## 5. Conclusions

In this study, an optimal robust state feedback controller is used to control biped robots walking in the lateral plane on slope. To this end, a biped robot is regarded and modeled in that plane. State feedback control is employed as a robust controller to control heavy nonlinear dynamic equations of the robot. Moreover, a multi-objective particle swarm optimization algorithm is used to design the parameters of the proposed controller. In the proposed controller, effectual techniques, such as a fuzzy based approach which prunes the archive, the turbulence operator that causes particles to escape straightforwardly, and Sigma method which finds the best personal positions of particles, are used. The results elucidate that the proposed method performs effectively in designing the parameters of the controller in comparison to three well-known algorithms, modified NSGAII, Sigma method, and MATLAB's Toolbox MOGA. Indeed, the proposed approach can be regarded as a promising approach to control various similar nonlinear systems, especially, biped robots. Furthermore, the normalized summation of angles errors and normalized summation of control effort are two conflicting objective functions. By using three points of the obtained Pareto front, six parameters of the controller are designed. The first point chosen in the Pareto front has the minimum normalized summation of angles errors, the third point has the minimum normalized summation of control effort, and the second point is the optimal point minimizing both objective functions simultaneously.

## Figures and Tables

**Table 1:** The anthropometric parameters of the model.

	First link	Second link	Third link	Unit
Mass	*m*_1_ = 13.75	*m*_2_ = 46.5	*m*_3_ = 13.75	kg
Inertia	*I*_1_ = 1.4	*I*_2_ = 3.25	*I*_3_ = 1.4	Kg·m^2^
Length	*l*_1_ = 0.91	*l*_2_ = 0.8	*l*_3_ = 0.91	m
CG	*h*_1_ = 0.50	*h*_2_ = 0.27	*h*_3_ = 0.50	m

**Table 2:** The objective functions and their associated design variables for the optimum points of Figure 2.

Optimum design point	A	B	C
Normalized summation of angles errors	1.22 × 10^−2^	4.14 × 10^−1^	8.23 × 10^−1^
Normalized summation of control efforts	9.67 × 10^−1^	5.27 × 10^−1^	1.09 × 10^−1^
Design variable *K*_1_	−1.80 × 10^2^	−8.33 × 10^1^	−8.44 × 10^1^
Design variable *K*_2_	−9.49 × 10^3^	−8.23 × 10^3^	−6.64 × 10^3^
Design variable *K*_3_	−1.90 × 10^2^	−1.11 × 10^2^	−1.09 × 10^2^
Design variable *K*_1_′	−9.39 × 10^3^	−5.00 × 10^3^	−4.89 × 10^3^
Design variable *K*_2_′	−1.80 × 10^2^	−3.91 × 10^−1^	−2.55 × 10^−2^
Design variable *K*_3_′	−9.00 × 10^2^	−6.49 × 10^−2^	−1.03 × 10^1^
